# Administration of antigenically distinct influenza viral particle combinations as an influenza vaccine strategy

**DOI:** 10.1371/journal.ppat.1012878

**Published:** 2025-01-22

**Authors:** Xinyu Zhu, Zhaochen Luo, Rebecca A. Leonard, Cait E. Hamele, Rachel L. Spreng, Nicholas S. Heaton

**Affiliations:** 1 Department of Molecular Genetics and Microbiology, Duke University School of Medicine, Durham, North Carolina, United States of America; 2 Duke Human Vaccine Institute, Duke University School of Medicine, Durham, North Carolina, United States of America; Dalhousie University, CANADA

## Abstract

One approach for developing a more universal influenza vaccine is to elicit strong immune responses against canonically immunosubdominant epitopes in the surface exposed viral glycoproteins. While standard vaccines typically induce responses directed primarily against mutable epitopes in the hemagglutinin (HA) head domain, there are generally limited or variable responses directed against epitopes in the relatively more conserved HA stalk domain and neuraminidase (NA) proteins. Here we describe a vaccine approach that utilizes a combination of wildtype (WT) influenza virus particles along with virus particles engineered to display a trimerized HA stalk in place of the full-length HA protein to elicit both responses simultaneously. After initially generating the “headless” HA-containing viral particles in the A/Hawaii/70/2019 (HI/19) genetic background and demonstrating the ability to elicit protective immune responses directed against the HA-stalk and NA, we co-formulated those virions with unmodified WT viral particles. The combination vaccine elicited “hybrid” and protective responses directed against the HA-head, HA-stalk, and NA proteins in both naïve and pre-immune mice and ferrets. Collectively, our results highlight a potentially generalizable method combining viral particles with differential antigenic compositions to elicit broader immune responses that may lead to more durable protection from influenza disease post-vaccination.

## Author summary

Influenza A virus infections remain a significant and persistent global challenge, as the viruses cause seasonal outbreaks and have pandemic-causing potential. Thus, improving the protection afforded by influenza vaccines is of high importance for human health. In this study we produced influenza viral particles that lack the classical immunodominant epitopes typically present in the head domain of the hemagglutinin (HA) protein. Vaccination with these modified virions allowed strong immune responses to develop against other protective viral epitopes, particularly within the relatively conserved HA stalk domain. We then showed that co-formulation of our engineered viral particles with unmodified virions allowed a “hybrid” vaccine response directed against all major viral epitopes of interest and provided protection from virally-induced disease. In ferrets with pre-existing influenza immunity, the experimental vaccine regimen also effectively elicited differential antibody profiles and allowed better control of infection in a heterologous viral challenge model. These data together suggest this approach has potential to serve as an improved, next-generation, influenza virus vaccine.

## Introduction

For the global community, influenza virus infections pose a perennial and significant threat. Outside of relatively minor seasonal disease, these viruses can cause severe illnesses in humans, resulting in hundreds of thousands of deaths worldwide every year [1,2]. One member of the influenza virus family, influenza A virus (IAV), is responsible for a significant proportion of this disease. IAV harbors its eight negative-sense, single-stranded genomic RNAs, encoding at least 10 viral proteins, in an enveloped viral particle that is efficiently transmitted via the respiratory route [3]. Hemagglutinin (HA), the most abundant viral surface glycoprotein on the viral particle, is the primary target for protective antibodies and consists of head and stalk domains. The globular HA head domain is responsible for sialic acid receptor binding and the HA stalk facilitates viral and cellular membranes fusion during infection [4]. Annual vaccination with the seasonal influenza vaccines represents a crucial public health measure for limiting disease. Most antibodies elicited by the seasonal vaccine target the HA head domain and are thought to function primarily by preventing viral infection via impeding HA-receptor binding [5–7]. These neutralizing antibodies offer robust protection against viral infection [8], however, immune pressure selects for mutations in the globular HA head that permit escape from antibody neutralization [9,10]. Thus, traditional vaccine-induced immunity is mostly limited to the specific strains included in the vaccine formulation.

Given this limitation, there is much interest in developing more universal influenza vaccines capable of eliciting protection against more antigenically diverse strains. Other viral glycoprotein domains that could theoretically be targeted to achieve this goal include the HA stalk domain. Structurally positioned just below the HA head domain, the HA stalk domain is generally more conserved from strain to strain, likely due to stringent structural constraints and generally reduced immune pressure [11,12]. Although stalk-directed antibodies are limited in their ability to directly neutralize virions, studies have demonstrated that monoclonal antibodies targeting specific epitopes in HA stalk have inhibitory activities [13,14]. For example, recognition of the HA stalk can induce antibody-dependent cellular cytotoxicity (ADCC) and confer protection during viral infection [15,16]. The magnitude of HA stalk-directed antibody responses induced by traditional vaccines is typically low, however, and the optimal approach for raising these antibodies remains an open question. Numerous experimental approaches have been previously reported, such as rationally designed peptides, recombinant HA stalk proteins, self-assembling nanoparticles, chimeric HAs, virus-like particles, and differentially glycosylated HA proteins [17–30]. In addition to the HA stalk, the neuraminidase (NA) protein, the second most abundant glycoprotein, is another attractive antigen for generating more protective/durable immune responses. Studies have demonstrated that NA-directed antibody responses provide good protection efficacy in animal models [31–36], and NA is also generally more conserved than the HA head domain.

Outside of selecting the antigens/epitopes of interest that will serve as the basis for more universal protection from influenza disease, the modality of their production and delivery also remains an open question. As influenza viral particles harbor the full complement of viral structural proteins and are immunogenic without additional adjuvating compounds, next-generation vaccine strategies centered on influenza virus particles have garnered some interest. Previous studies have reported influenza viruses presenting chimeric or mutant HAs can induce protective levels of stalk-directed antibodies [37–42], typically after a prime/boost regimen. Additionally, our laboratory has designed influenza virus particles based on a laboratory-adapted strain A/Puerto Rico/8/34 (PR8) strain that either completely lack an HA protein [33] or harbor a trimerized, headless HA stalk antigen to elicit immune responses primarily against the NA or NA and HA stalk proteins, respectively [43]. Absent the normally immunodominant HA head domain, these virus particle-based vaccines have been found to be effective at eliciting immune responses to protective, non-head epitopes [33,43].

While our previous work has shown that vaccination with a headless HA-containing viral particle can elicit strong stalk-directed responses and provide protection from disease, the “best-case” scenario would be for a vaccine to simultaneously elicit HA-head directed responses as well. Furthermore, all of our previous work has been done in a laboratory adapted strain, preventing an understanding of the potential adaptability of this approach to clinically relevant viral strains. To address these two major limitations, here we designed an HA stalk protein derived from a contemporary H1N1 virus strain, A/Hawaii/70/2019 (HI/19) and incorporated it onto the surface of an authentic HI/19 virus particle. Following vaccination with the headless HA particle vaccine, we again observed that high-magnitude and protective HA stalk and NA-directed antibody responses were elicited. We next co-formulated this headless HA viral particle with unmodified WT HI/19 viral particles to produce a combination vaccine with multiple HA protein configurations. Vaccination of mice and ferrets with the combination vaccine elicited high, and functionally protective, levels of HA stalk-directed and HA head antibodies, as well as NA antibodies. Importantly, we found that these vaccine responses could be elicited in both naïve and pre-immune animals. Thus, combinations of engineered viral particles do not appear to compete when co-administered and could potentially be used to elicit the specific types of immune responses predicted to provide enhanced protection from influenza disease, regardless of influenza infection history.

## Results

### Generation of an HI/19 based, headless HA viral particle vaccine

**Fig 1:**
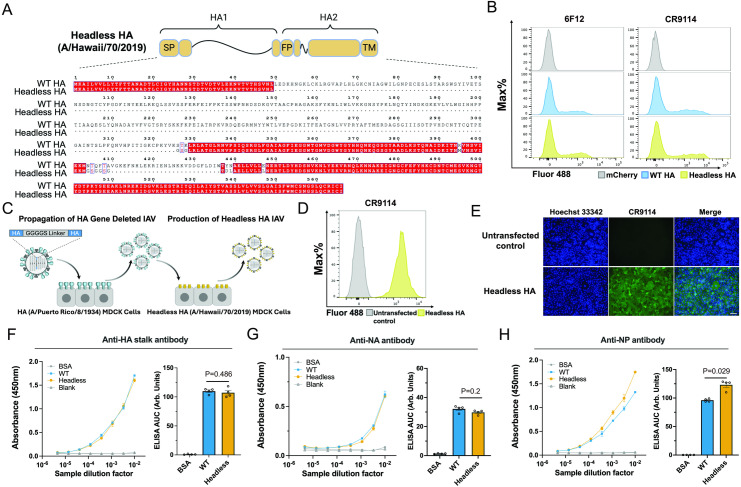
Design and generation of a contemporary H1N1 headless HA influenza virus. **(A)** The design of the headless HI/19 HA construct. SP, signal peptide. FP, fusion peptide. TM, transmembrane domain. S_326_T_327_K_328_ and N_404_T_405_Q_406_F_407_T_408_ were replaced by GSG and GSGGSG linkers, respectively. K_395_M, Y_438_D, N_439_L and E_447_L mutantions increased internal stabilization. White font on red background represents unchanged residues; dots represent deletions; clear boxes with red font indicates conservative mutantions with amino acids that have similar properties; non-boxed mutations indicate nonconservative mutantions. **(B)** Recognition of the headless HI/19 HA construct in HEK-293T via flow cytometry. HEK-293T transfected with WT HI/19 HA or the headless HI/19 HA plasmids and after 24 h cells were collected and stained with HA stalk-directed mouse antibody 6F12 (1:50 dilution) and human antibody CR9114 (1:500 dilution). **(C)** Diagram of the approach to generate the headless HI/19 virus. An image available at https://bioart.niaid.nih.gov/bioart/432 provided by the NIH BioArt source was used in this panel. **(D)** Flow cytometry of the MDCK cell line stably expressing headless HI/19 HA. **(E)** Staining of the cell line stably expressing the headless HI/19 HA via immunofluorescence microscopy. Scale bar, 100 μm. Blue, nuclei; Green, HA stalk. **(F)** HA protein level comparison between WT HI/19 vaccine and normalized headless HI/19 vaccine via ELISA. N = 4 technical replicates. **(G)** NA protein level comparison between WT HI/19 vaccine and normalized headless HI/19 vaccine via ELISA. N = 4 technical replicates. **(H)** NP protein level comparison between WT HI/19 vaccine and normalized headless HI/19 vaccine via ELISA. N = 4 technical replicates. All experiments were performed at least two times and similar results were observed. For panels F–H, Mann-Whitney U tests were performed. Data shown as mean ± the standard error of the mean (SEM).

To develop our vaccine, we initially designed a headless HA antigen in the background of the contemporary H1 HA derived from the H1N1 strain HI/19. Based on previous reports [22], the HA1 region and the membrane distal HA1 and HA2 regions in HI/19 HA were deleted or truncated. Next, we introduced a glycine-rich linker and internal mutations to enhance stalk folding and stabilization (Fig 1A). To assess HA protein expression, trafficking, and folding, plasmids encoding either the headless HI/19 HA or wild-type (WT) HI/19 HA were transfected into HEK-293T cells. The well characterized monoclonal antibodies 6F12 and CR9114 [13,44] bound both groups of transfected cells to similar extents, suggesting the correct expression and folding of the headless HA protein in mammalian cells (Fig 1B).

To produce headless HA antigen containing virus particles (Fig 1C), we first needed to generate an MDCK cell line stably expressing the headless HI/19 HA protein. A lentivirus-based approach was used to introduce the gene and expression of the HI/19 protein was confirmed via flow cytometry and immunofluorescence assay (Fig 1D and 1E). Next, we modified the reverse genetics system for WT HI/19 so that the HA coding region in segment four of the viral genome was replaced with a short GGGGS peptide. This HA deletion virus was then rescued in HEK-293T cells by introducing the eight vRNA encoding plasmids along with an HA protein expression plasmid; this “first-round” virus was then propagated on a previously established HA (PR8) MDCK cell line [33]. To produce the headless HA HI/19 viral particles themselves, the HA deletion virus was used to infect the headless HA MDCK cell line at a high MOI and the progeny virions were collected from the supernatant. Viral particles were then purified from the supernatant by ultracentrifugation and subsequently inactivated. Because the control WT HI/19 virus was replication competent, we infected unmodified MDCK cells to collect viral particles and inactivated them as per the approach for the headless HA viral particles. After normalizing vaccines based on HA-stalk antibody binding in an ELISA assay, we measured the NA and nucleoprotein (NP) content. While NA levels were indistinguishable between the two viruses, NP levels were higher in the headless virus suggesting the glycoprotein density on the headless HA viral particles was slightly lower than WT virions (Fig 1F–1H).

### The headless HA HI/19 vaccine is immunogenic and provides protection from challenge in naïve mice

**Fig 2:**
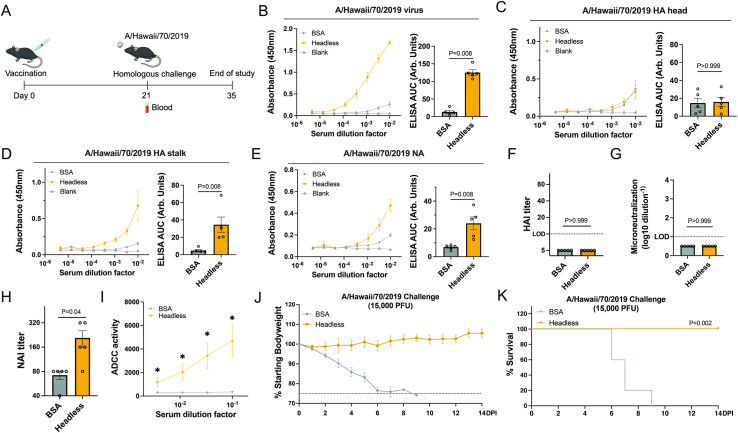
Immunogenicity and protective efficacy of the headless HA HI/19 vaccine in mice. **(A)** Schematic of the vaccination regimen and challenge. Images available at https://bioart.niaid.nih.gov/bioart/279, https://bioart.niaid.nih.gov/bioart/505, https://bioart.niaid.nih.gov/bioart/87 and https://bioart.niaid.nih.gov/bioart/187 provided by the NIH BioArt source were used in this panel. **(B–E)** Sera from the two groups, including headless HI/19 and BSA, collected at 21 days post-vaccination was used to detect antibody responses against whole HI/19 virus **(B)**, HI/19 HA head **(C)**, HA/19 HA stalk **(D)** and HA/19 NA **(E)** via ELISA. N = 5 mice. **(F)** Hemagglutination inhibition assay (HAI) with sera from two groups of mice. HAI titers were calculated as the reciprocal of the last dilution of sera that inhibited hemagglutination. LOD = 10. N = 5 mice. **(G)** Microneutralization assay with sera from two groups of mice. Limit of detection (LOD) = 1. N = 5 mice. **(H)** Neuraminidase-inhibition (NAI) assay with sera from two groups of mice. NAI titer is calculated as the reciprocal of the lowest dilution of sera that inhibited at least 50% NA activity. N = 5 mice. **(I)** Antibody dependent cellular cytotoxicity (ADCC) assay with sera from two groups of mice. N = 5 mice. *, p < 0.05. **(J)** Body weight of vaccinated mice from two groups was monitored until 14 days post infection. N = 5 mice. **(K)** Survival of vaccinated mice from two groups was monitored until 14 days post infection. N = 5 mice. Undetectable samples were treated as 50% of LOD for statistical analysis. All experiments were performed at least 2 times and similar results were observed. For panels B-H, Mann-Whitney U tests were performed. For panels I, FDR adjusted p-values from Wilcoxon rank-sum exact tests to control for multiple comparisons were shown. For panel K, log-rank (Mantel-Cox) tests were performed. Data shown as mean ± SEM.

To understand the immunogenicity of the headless HA viral particles, naïve mice were vaccinated with a single dose of the headless HI/19 vaccine or the control protein BSA (Fig 2A). We first measured sera reactivity against intact WT HI/19 viral particles and observed a strong response uniquely in the viral vaccine group (Fig 2B). To better understand the composition of that response, we next performed ELISAs with purified HI/19 HA head, HA stalk, and NA proteins. As expected, no measurable response against the HI/19 HA head domain was detected (Fig 2C). We did detect however, a response that was significantly above background for both the HA stalk and NA protein (Fig 2D and 2E).

To assess the functionality of the viral glycoprotein-reactive antibodies, we first measured the ability of the sera to mediate hemagglutinin inhibition (HAI) and microneutralization, as these are the standard assays used to benchmark a protective immune response. Unsurprisingly, because these activities are mediated predominantly by HA head-reactive antibodies, we did not detect any signal above background (Fig 2F and 2G). A NA inhibition (NAI) assay demonstrated activity in headless HA HI/19 post-vaccination sera as expected due to the presence of NA on the headless viral particles (Fig 2H). Finally, we tested for antibody-dependent cellular cytotoxicity (ADCC) activity, as this is a known activity of HA stalk-directed antibodies [15,16] and found that sera from the headless HI/19 group showed significant ADCC activity (Fig 2I). Thus, at least some of the sera antibodies that can bind the viral glycoproteins in ELISA have functional activity. To understand if the types of immune responses elicited by the headless HA viral vaccine could protect from a lethal challenge, vaccinated animals were challenged with the homologous HI/19 strain. Despite no measurable head-directed antibodies, we observed complete protection from body weight loss and mortality in headless HI/19 group, while all mice in BSA group succumbed to infection (Fig 2J and 2K).

### Combination of hveadless HA and WT HI/19 vaccines elicit hybrid responses

Although the headless HA HI/19 vaccine had demonstrated protective efficacy, we next wanted to understand if we could devise a vaccine formulation that would also elicit an HA-head directed response without compromising the HA-stalk and NA directed responses we previously observed. Previous work has shown that, at least with purified protein antigens, physical attachment of the head and stalk domain are required for the HA head immunodominance phenomenon [45]. We therefore hypothesized that if we were to co-formulate WT viral particles with our headless HA viral particles, we could potentially elicit a hybrid response wherein strong responses to the HA stalk, HA head, and NA proteins would be raised post-vaccination.

**Fig 3:**
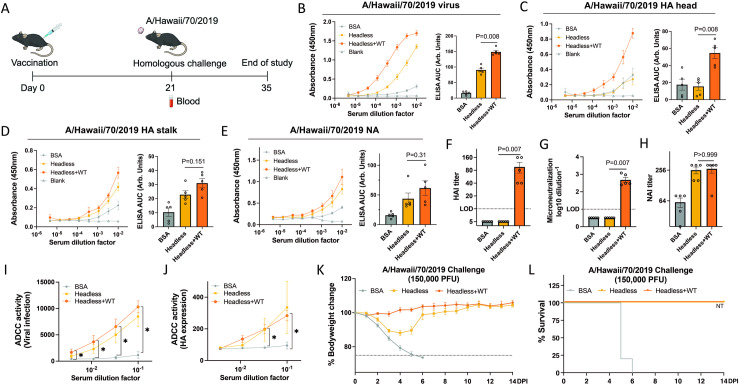
Immunogenicity and protective efficacy of headless HA+WT HI/19 vaccine in mice. **(A)** Schematic of the vaccination regimen. Images available at https://bioart.niaid.nih.gov/bioart/279, https://bioart.niaid.nih.gov/bioart/505, https://bioart.niaid.nih.gov/bioart/87 and https://bioart.niaid.nih.gov/bioart/187 provided by the NIH BioArt source were used in this figure panel. **(B–E)** Sera from the three groups, including headless HA+WT HI/19, headless HI/19 and BSA, collected at 21 days post-vaccination was used to detect antibody responses against whole HI/19 virus **(B)**, HI/19 HA head **(C)**, HI/19 HA stalk **(D)** and HI/19 NA **(E)** via ELISA. N = 5 mice. **(F)** Hemagglutination inhibition assay (HAI) with sera from three groups. HAI titers were calculated as the reciprocal of the last dilution of sera that inhibited hemagglutination. LOD = 10. N = 5 mice. **(G)** Microneutralization assay with sera from three groups. LOD = 1. N = 5 mice. **(H)** Neuraminidase-inhibition (NAI) assay with sera from three groups collected at 21 days post-vaccination. NAI titer is calculated as the reciprocal of the lowest dilution of sera that inhibited at least 50% NA activity. N = 5 mice. **(I)** Antibody dependent cellular cytotoxicity (ADCC) assay on virally infected cells with sera from three groups collected at 21 days post-vaccination. N = 5 mice. *, p < 0.05. **(J)** ADCC assay as in I, but with target cells only expressing the HA protein. *, p < 0.05. **(K)** Body weight of vaccinated mice from the three vaccines groups was monitored until 14 days post infection. N = 5 mice. **(L)** Survival of vaccinated mice from three groups was monitored until 14 days post infection. N = 5 mice. NT, not tested. Undetectable samples were treated as 50% of LOD for statistical analysis. All experiments were performed at least 2 times and similar results were observed. For panels B-H, Mann-Whitney U tests were performed. For panels I and J, FDR adjusted p-values from Wilcoxon rank-sum exact tests to control for multiple comparisons were shown. Data shown as mean ± SEM.

As a first test of this hypothesis, we mixed the headless HA viral particles and WT viral particles 1:1 (based on HA content), and vaccinated mice with either the combination headless HA+WT HI/19 vaccine, headless HI/19 vaccine alone, or control BSA protein (Fig 3A). ELISAs using post-vaccination sera revealed that levels of antibodies reactive against whole HI/19 virus were significantly increased in the headless HA+WT HI/19 group compared to the headless HI/19 group (Fig 3B). Subsequent ELISAs against individual proteins/domains of the glycoproteins revealed that compared to the headless HI/19 group, the headless HA+WT HI/19 group displayed similar levels of HA stalk and NA-directed antibodies while adding an HA head directed response (Fig 3C–3E). Functional antibody assays demonstrated that HAI and viral neutralization activity were uniquely detectable in the combination headless HA+WT HI/19 vaccine group (Fig 3F and 3G), while sera ADCC and NAI levels in the headless HA+WT HI/19 group were similar to those found in the headless HI/19-only group (Fig 3H and 3I). To ensure that HA-stalk binding antibodies were mediating at least some of the observed ADCC, we repeated the assay with target cells that only express the HA protein and observed similar results (Fig 3J). To assess the protection afforded by these vaccines, the vaccinated mice were challenged with the homologous vaccine strain (HI/19), using a higher dose than used in the previous experiments. While mice in the headless HA+WT HI/19 and headless HI/19 groups both survived, mice in the headless HA+WT HI/19 group experienced significant body weight loss (Fig 3K and 3L). These results indicate that the headless HA+WT HI/19 vaccine can elicit “hybrid” HA head, stalk, and NA-directed responses that provide improved protection from high-dose challenge relative to the headless HA vaccine alone.

### Headless HA+WT HI/19 vaccine enhances antibody responses against conserved viral epitopes in mice with pre-existing immunity

**Fig 4:**
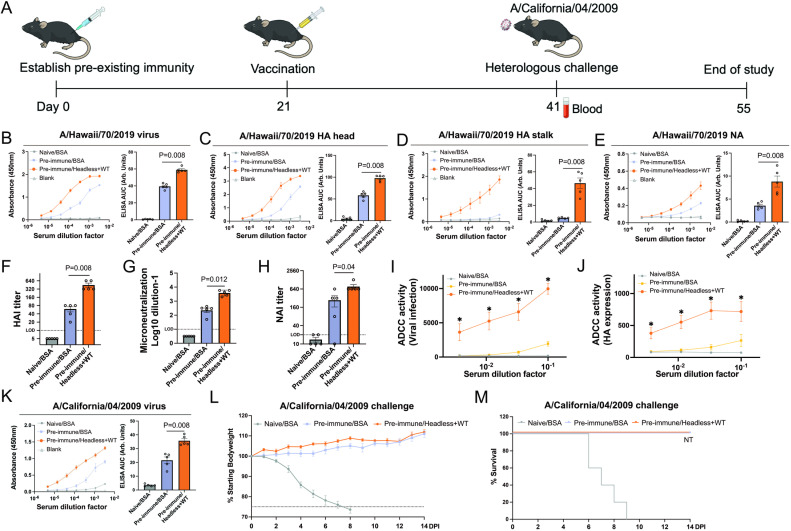
Immunogenicity and protective efficacy of headless HA+WT HI/19 vaccine in mice with pre-existing immunity. **(A)** The schematic for establishment of pre-existing immunity followed by vaccination. Images available at https://bioart.niaid.nih.gov/bioart/279, https://bioart.niaid.nih.gov/bioart/505, https://bioart.niaid.nih.gov/bioart/506, https://bioart.niaid.nih.gov/bioart/87, and https://bioart.niaid.nih.gov/bioart/187 provided by the NIH BioArt source were used in this panel. **(B–E)** Immune sera was used to detect antibody responses against whole HI/19 virus **(B)**, HI/19 HA head **(C)**, HI/19 HA stalk **(D)** and HI/19 NA **(E)** via ELISA. N = 5 mice. **(F)** Hemagglutination inhibition assay (HAI) with immune sera. HAI titers were calculated as the reciprocal of the last dilution of sera that inhibited hemagglutination. LOD = 10. N = 5 mice. **(G)** Microneutralization assay with immune sera. LOD = 1. N = 5 mice. **(H)** Neuraminidase-inhibition (NAI) assay with post-boost sera. NAI titer is calculated as the reciprocal of the lowest dilution of sera that inhibited at least 50% NA activity. N = 5 mice. (I) Antibody dependent cellular cytotoxicity (ADCC) assay against infected cells with post-vaccination sera. N = 5 mice. *, p < 0.05. **(J)** ADCC assay targeting cells only express the viral HA protein. *, p < 0.05. **(K)** Post-boost sera were used to detect antibody responses against whole A/California/04/2009 virus via ELISA. N = 5 mice. **(L)** Body weight of vaccinated mice with pre-existing immunity was monitored for 14 days post infection. N ≥ 4 mice. **(M)** Survival of vaccinated mice with pre-existing immunity was monitored for 14 days post infection. N ≥ 4 mice. NT, not tested. Undetectable samples were treated as 50% of LOD for statistical analysis. All experiments were performed at least 2 times and similar results were observed. For panels B-H and K, Mann-Whitney U tests were performed. For panels I and J, FDR adjusted p-values from Wilcoxon rank-sum exact tests to control for multiple comparisons were shown. Data shown as mean ± SEM.

Given most humans over the age of one have pre-existing immunity to influenza virus elicited by natural infections or seasonal vaccines, we sought to evaluate the efficacy of our headless HA+WT HI/19 vaccine in mice with pre-existing immunity. To establish pre-existing immunity, mice received one administration of inactivated WT HI/19 viral particles. Animals were then vaccinated with either BSA or the combination headless HA+WT HI/19 vaccine (Fig 4A). Antibody levels in post-vaccination sera were then analyzed via ELISA. As expected, antibody responses to WT viral particles were improved post-vaccination compared to BSA control, even in pre-immune animals (Fig 4B). Partially deconvoluting that aggregate reactivity, we found that antibodies against the HA head, HA stalk, and NA were all enhanced by the headless HA+WT HI/19 vaccine (Fig 4C–4E). Importantly, HA stalk-directed antibody responses were still induced by the headless HA+WT HI/19 vaccine (Fig 4D). Finally, we vaccinated another group of pre-immune mice with only WT HI/19 viral particles to compare to the vaccine groups described above. As expected, the headless HA+WT HI/19 group showed uniquely improved HA stalk- and NA-directed antibody responses, but no difference in HA head-directed antibodies when compared with the WT HI/19 vaccine group (S1 Fig). These data are consistent with a novel and unique immune profile after administration of the combination vaccine.

Assays to define antibody functionality of the headless HA+WT HI/19 vaccine group demonstrated that HAI, viral neutralization, and NAI were all improved after vaccination of pre-immune animals (Fig 4F–4H), as was the ADCC activity against both infected and HA-only expressing cells (Fig 4I and 4J). Finally, to understand if the antibodies elicited by this vaccine scheme had the potential to recognize antigenically distinct viruses, we performed ELISAs against the heterologous, but homosubtypic, A/California/04/2009 virus. We observed significantly increased antibody binding in pre-immune animals that had been vaccinated with the headless HA+WT vaccine, demonstrating the potential of the induced antibodies to recognize heterologous viruses (Fig 4K). To test the ability of those antibodies to protect from disease, we next challenged mice with a lethal dose of the A/California/04/2009 virus. While the control animals all succumbed to infection, the established pre-existing immunity occluded our ability to measure the effects of our vaccine, as animals from both groups were completely protected from weight loss and mortality (Fig 4L and 4M).

### The headless HA+WT HI/19 vaccine enhances antibody responses in ferrets with pre-existing immunity, providing protection against heterologous virus infection

**Fig 5:**
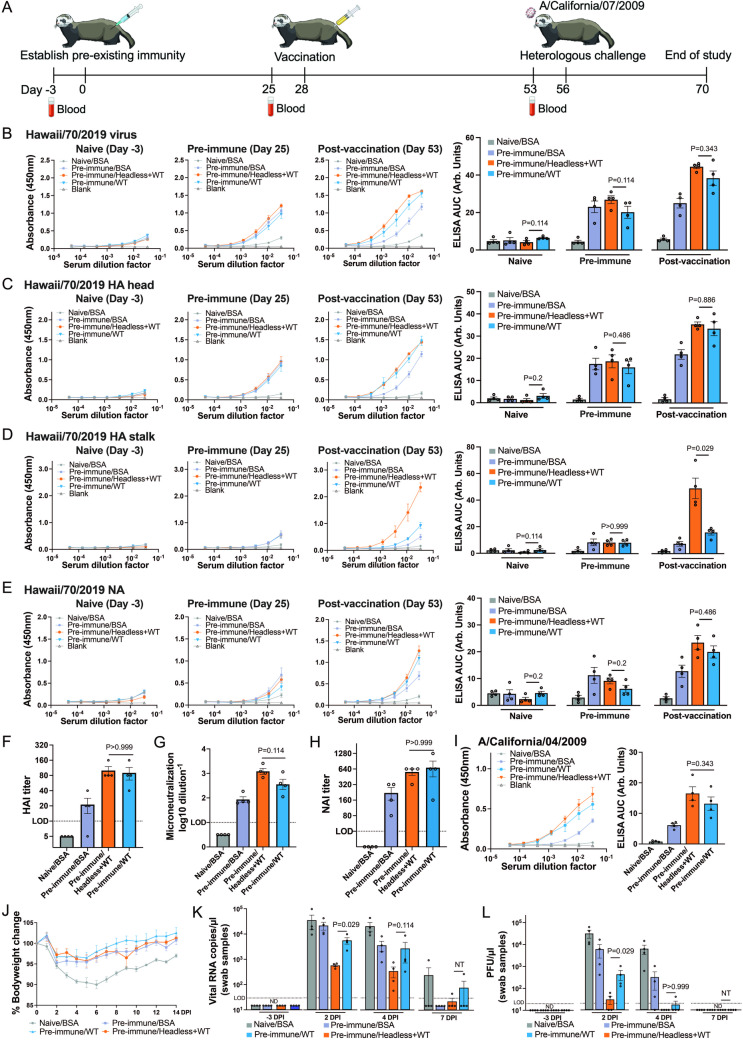
Immunogenicity and efficacy of the headless HA+WT HI/19 vaccine in ferrets with pre-existing immunity. **(A)** Schematic of the vaccine regimen. Ferrets received one dose of WT HI/19 vaccine or BSA to establish pre-existing immunity. After 28 days, ferrets were administered either BSA, WT HI/19 vaccine or the headless HA+WT HI/19 vaccine. After another 28 days, ferrets were challenged with A/California/07/2009 virus. All sera were collected 3 days before vaccination or virus challenge. Images available at https://bioart.niaid.nih.gov/bioart/150, https://bioart.niaid.nih.gov/bioart/505, https://bioart.niaid.nih.gov/bioart/506, https://bioart.niaid.nih.gov/bioart/87 and https://bioart.niaid.nih.gov/bioart/187 provided by the NIH BioArt source were used in this figure panel. **(B–E)** Naive sera collected before vaccination, sera collected after establishment of pre-existing immunity, and sera collected post-vaccination were used to detect antibody responses against whole HI/19 virus. **(B)**, HI/19 HA head **(C)**, HI/19 HA stalk **(D)** and HI/19 NA **(E)** via ELISA. N = 4 ferrets. **(F)** Hemagglutination inhibition assay (HAI) with post-vaccination sera. HAI titers were calculated as the reciprocal of the last dilution of sera that inhibited hemagglutination. LOD = 10. N = 4 ferrets. **(G)** Microneutralization assay with post-vaccination sera. LOD = 1. N = 4 ferrets. **(H)** Neuraminidase-inhibition (NAI) assay with post-vaccination sera. NAI titer is calculated as the reciprocal of the lowest dilution of sera that inhibited at least 50% NA activity. N = 4 ferrets. LOD = 40. **(I)** Post-vaccination sera was used to detect antibody responses against whole A/California/04/2009 virus via ELISA. N = 4 ferrets. **(J)** Body weight of vaccinated ferrets with pre-existing immunity was monitored until 14 days post infection. N = 4 ferrets. **(K)** Viral RNA level in nasal swab samples after infection. Nasal swab samples collected at 3 days before infection, 2 days post-infection (DPI), 4 DPI, 7 DPI were analyzed for RT-qPCR. Viral NP gene was detected. **(L)** Plaque assay in nasal swab samples after infection. Nasal swab samples were collected at 3 days before infection, 2 DPI , 4 DPI, 7 DPI were analyzed. Undetectable samples were treated as 50% of LOD for statistical analysis. ND, not detected. NT, not tested. Except for panels F-J, all experiments were technically performed at least 2 times and similar results were observed. For panels B-I and K-L, Mann-Whitney U tests were performed. Data shown as mean ± SEM.

While the headless HA+WT HI/19 combination vaccine was effective in eliciting differential antibody profiles in mice, we were ultimately unable to demonstrate improved protection in pre-immune animals after heterologous challenge. We therefore decided to evaluate the performance of our vaccine in the ferret model, which is generally considered a better model of human vaccine responses and clinical disease [46,47]. We administered WT HI/19 viral particles to animals to establish pre-existing immunity and then vaccinated with either BSA, the headless HA+WT HI/19 vaccine, or WT HI/19 viral particles to mimic administration of existing vaccines (Fig 5A).

We next conducted experiments using ferret sera that was collected (1) prior to any viral exposure, (2) post-establishment of pre-existing immunity, or (3) post-vaccination. ELISAs demonstrated that all ferrets exhibited low baseline immunity against the HI/19 virus and that the initial dose of WT HI/19 vaccine established similar levels of pre-existing immunity across all of the groups (Fig 5B–5E). Following vaccination, the headless HA+WT HI/19 and the WT-only group displayed similarly increased levels of antibodies against the HI/19 viral particles, the HA head, and the NA protein (Fig 5B–5E). The headless HA+WT HI/19 group, however, uniquely showed a substantial HA stalk-directed antibody response (Fig 5D). HAI, viral neutralization, and NAI activities in the post-vaccination sera showed that both the headless HA+WT HI/19 and WT only vaccines were both effective in improving pre-immune sera activities, as expected from the ELISA analysis (Fig 5F–5H). Due to a lack of ferret-specific reagents, however, we were unable to measure ADCC activity. Thus, similar to what was observed in mice, the headless HA+WT HI/19 vaccine can simultaneously induce responses to classic immunodominant epitopes along with responses to subdominant epitopes in ferrets, at least to the extent to which we could measure them.

Next, we wanted to understand whether the increased breadth of antibodies would provide increased protection during heterologous virus challenge. We initially measured sera binding to WT A/California/04/2009 viral particles and found both the headless HA+WT HI/19 and WT HI/19-only groups displayed improved antibody reactivity compared to the pre-immune only animals. Although we observed a slight trend towards higher reactivity in the headless HA+WT group, it was not statistically significant (Fig 5I). Despite similar overall reactivity, we challenged the animals with the heterologous A/California/07/2009 virus. While the BSA-only control group lost ~10% of their pre-infection bodyweight, the three other groups showed reduced body weight loss of similar magnitudes, demonstrating some degree of protection from clinical disease mediated by pre-existing immunity (Fig 5J). Analysis of viral RNA load in nasal/oral swab samples collected at 2- and 4-days post-infection, however, revealed differential ability of the vaccinated animals to control viral replication. Notably, at 2-days post-infection, the headless HA+WT HI/19 group had significantly reduced viral RNA levels compared to the pre-immune/WT group (Fig 5K). Further, plaque assays for infectious virus showed a similar trend at 2- and 4-days post-infection where viral titer in the headless HA+WT HI/19 group was significantly decreased compared with the pre-immune/WT group and ultimately cleared from the animals with quicker kinetics (Fig 5L). These data suggest that the antibody profile with a uniquely strong HA stalk-directed component (as elicited by the combination headless HA+WT HI/19 vaccine) provides additional advantages during infection.

## Discussion

There is high interest in the development of more durable and universally protective influenza vaccines. One promising approach is to simultaneously elicit immune responses to multiple viral glycoprotein epitopes. Many experimental vaccine approaches attempt to elicit responses targeting more conserved viral epitopes (e.g. in the HA stalk) often at the expense of responses targeting protective, but evolutionary flexible, epitopes (e.g. classical antigenic sites in the HA head). In this study, we demonstrated that an approach of mixing virions that display different antigenic properties can simultaneously elicit a “hybrid” antibody response that recognizes epitopes in the HA head, stalk, and NA. Due to the lack of antigenic competition between the different viral particles, this approach may represent a practical path forward to add additional immune responses to current vaccine approaches instead of replacing them.

HA stalk-directed antibodies are generally less potent than HA head-directed antibodies due to their weak neutralizing activity [5,48,49]. These antibodies, however, can mediate effects via other mechanisms, such as ADCC [15,16]. Indeed, we noted uniquely enhanced ADCC activity in our headless HA+WT HI/19 vaccine group. Granulocytes, such as natural killer cells and neutrophils, expressing FcγRIII, can target infected cells by recognizing the Fc domain of specific IgG subtypes and trigger apoptosis in infected cells [50]. This non-neutralizing antiviral function is appreciated to provide protection against different viral infections [51–54]. Although antibodies against multiple viral proteins, such as HA stalk, NA, and NP, have all been reported to induce ADCC activity [15,55,56], the enhanced HA stalk-directed antibodies elicited by our headless HA+WT HI/19 vaccine likely drive much of the enhanced ADCC activity observed by the combination vaccine as enhanced responses are observed when target cells express only HA. We, however, did not formally measure the contributions of antibodies targeting different viral proteins. In addition to the sera ADCC activity measured in our study, antibody-dependent cell-mediated phagocytosis (ADCP), complement-dependent cytotoxicity (CDC) and steric inhibition of proteins in viral replication are also important inhibitory mechanisms for non-neutralizing antibodies [57,58]. Whether these activities were enhanced by the headless HA+WT HI/19 vaccine to similar degrees as the ADCC activity, however, remains an open question. In addition to causing infected cell death, crosslinked effector cells can secrete antiviral proteins, such as IFNγ, TNFα, and MIP1α, creating an antiviral environment [59,60]. A combination of these two activities likely explains why we observed the lowest viral RNA load and viral titers in ferrets vaccinated with headless HA+WT HI/19 vaccine, yet similar overall serum viral neutralization ability compared to the pre-immune/WT HI/19 group.

Despite the success of our vaccine in eliciting an antibody response that recognizes more viral protein epitopes, our study has limitations. First, we did not test the protection mediated by our vaccine against non-H1N1 viruses. While we predict that our H1N1-based vaccine would provide some recognition of at least other group 1 HA viruses, the amount of recognition and whether it provides any meaningful protection remain unclear. Additionally, in our experimental mouse and ferret models, we observed either similar or modestly improved protection from challenge despite significantly improved HA stalk-directed responses when HA head-directed responses were present. Whether the magnitude of the HA stalk-directed responses needs to be further enhanced, or if other challenge models that more closely mimic human immune responses need to be used, remains unclear. Further, we did not test the levels of antibodies that recognize other viral proteins or CD8+ T-cell responses. It is possible these responses could be induced by this vaccine regimen and may contribute to protection, and it will be important to measure them in future studies. It is also worth noting that the complexity of human influenza pre-existing immunity (including the responses generated via natural infection) was not fully recapitulated in our animal models and may ultimately lead to differential vaccine-elicited responses. Finally, it will be also important to implement the headless HA+WT vaccine approach to other clinically relevant strains, including H3N2 and influenza B viruses, to investigate if this technology is broadly applicable in generating vaccines for diverse influenza viruses.

In conclusion, our combination virion-based vaccine in the background of a contemporary H1N1 strain increased levels of antibodies recognizing the relatively conserved HA stalk and NA protein while maintaining HA head-directed antibody responses compared to a WT viral particle-based approach. We could observe these responses in both naïve and pre-immune mice and ferrets, and the vaccine improved protection and/or mediated more rapid viral clearance depending on the experimental model used. Future work using these or similar approaches may represent a way to improve the breadth and/or durability of influenza vaccines using similar vaccine manufacturing, formulation, and distribution methods as current seasonal vaccines.

## Materials and methods

### Ethics statement

All mouse experiments were performed according to protocol A142-21-07, and all ferret experiments were performed according to protocol A077-23-03. Both protocols were approved by the Duke University Institutional Animal Care and Use Committee.

### Cell lines

HEK-293T and MDCK cells were both obtained from American Type Culture Collection (ATCC). HEK-293T cells were cultured with DMEM supplemented with 5% FBS, 1×Glutamax and 1% Penicillin/Streptomycin. MDCK cells were cultured in MEM supplemented with 5% FBS, 20 mM HEPES, 0.15% sodium bicarbonate and 1×Glutamax. HA (PR8) MDCK cell line was described in a previous study [33]. To generate the headless HI/19 HA MDCK cell line, the headless HI/19 HA sequence was designed based on previous study [22]. Briefly, the head HA1 region, membrane distal HA1 and HA2 regions in HI/19 HA were deleted or truncated and the headless HI/19 fragment was further synthesized at IDT and cloned into lentivirus vector pLex. The lentivirus expressing the headless HI/19 HA was transduced into WT MDCK cells. After 6 μg/ml puromycin selection and sorting using fluorescence activated cell sorting (FACS), the polyclonal headless HI/19 HA MDCK cells were further diluted in a 96-well plate and single cell clone was expanded and verified with CR9114 antibody. All cells were culture at 37 °C in incubator with 5% CO_2_.

### Headless HI/19 HA virus generation and vaccine preparation

A bicistronic pDZ rescue plasmid system from the HI/19 strain background was used to rescue the HA negative HI/19 virus. Firstly, the middle coding region of segment 4 HA pDZ was replaced with a GGGGS linker sequence to generate segment 4 HAps-GGGGS-HAps pDZ. A similar strategy was described in previous study [33]. Next, 0.5 μg HAps-GGGGS-HAps pDZ, 0.5 μg pLex-HA and 0.5 μg of the other seven viral segments in pDZ were co-transfected into HEK-293T cells. After 48 h, the supernatant was collected and inoculated into HA (PR8) MDCK cell line. A single plaque was expanded to create a plaque-purified HA negative HI/19 virus.

To generate the headless HI/19 vaccine, HA-negative HI/19 virus was propagated in HA (PR8) MDCK cells with post-infection media (Opti-MEM with 0.35% BSA, 0.01% FBS, 1 μg/ml TPCK). The supernatant was collected 72 hours post-infection (hpi) and further inoculated into headless HI/19 MDCK cells with 5 MOI. After a 1 h incubation, the infected headless HI/19 MDCK cells were washed with PBS and post-infection media (Opti-MEM with 0.35% BSA, 0.01% FBS, 1 μg/ml TPCK) was added. The supernatant containing headless HI/19 HA virus was collected 48 hpi. For WT HI/19 virus, it was propagated in regular MDCK cells. Headless HI/19 HA virus and WT HI/19 virus were further concentrated using a 30% (w/v) sucrose cushion via ultracentrifugation. Concentrated virus was inactivated with 0.02% formalin for 48 hours at 4 °C and dialyzed using Slide-A-Lyzer cassettes (Thermo Fisher, #66370). For vaccine formulation, inactivated virus was quantified using Pierce Rapid Gold BCA Protein Assay Kit (Thermo Fisher, #A53226). After normalization of the two vaccines by HA stalk levels, 2 μg WT HI/19 virus or 3.6 μg normalized headless HI/19 virus was diluted in pharmaceutical-grade PBS to a final volume of 50 μl (Corning, 21-040-CV) and, along with 50 μl AddaVax (InvivoGen, vac-adx-10) was vaccinated intramuscularly in the left hind leg of mouse. For ferret experiment, 5 μg WT HI/19 virus or 9 μg normalized headless HI/19 virus was administered intramuscularly. Normalized inactivated virus was mixed with an equal value of AddaVax (Invivogen, #vac-adx-10) to generate vaccine for vaccination.

### Experimental animal models

For all mouse experiments, six-week-old C57BL/6 female mice were used. The BSA group was vaccinated with 2 μg BSA, the WT HI/19 group was vaccinated with 2 μg WT HI/19 virus and then the headless HI/19 HA group was vaccinated with normalized 3.6 μg headless HI/19 HA virus. The headless HA+WT HI/19 group was vaccinated with 2 μg WT HI/19 virus and normalized 3.6 μg headless HI/19 HA virus. To establish pre-existing immunity, 2 μg BSA or 2 μg WT HI/19 virus was used and then the normalized vaccine was given 21 days after. Sera was collected at indicated time point. All virus challenges were conducted 21 days post-vaccination. Following anesthesia with 80 ul ketamine-xylazine mixture, all mice were intranasally infected with 40 μL virus. For A/Hawaii/70/2019 strain, 15000 PFU or 150000 PFU was used. For A/California/04/2009 strain, 100000 PFU was used. After infection, mice were weighed daily for 14 days. 25% body weight loss was treated as a humane endpoint.

For the ferret model, six-month-old male ferret were used. To establish pre-existing immunity, 5 μg BSA or 5 μg WT HI/19 virus were administered intramuscularly. After 28 days, 5 μg BSA, 5 μg WT HI/19 virus or 5 μg WT plus normalized 9 μg headless HI/19 HA virus was applied for vaccination. After an additional 28 days, ferrets were challenged with A/California/07/2009 strain (1000000 PFU). Ferrets were weighed daily for 14 days. Sera and nasal swab samples were collected at the indicated time points. All processes were permitted by the Duke University IACUC.

### Flow cytometry

HEK-293T cells were transfected with pLex-mCherry, pLex-PR8 HA or pLex-Headless HI/19 HA using Lipofectamine 2000 transfection reagent (Thermo Fisher, #11668019). After 24 h cells were trypsinized and washed with PBS containing 1% BSA. The suspension samples were incubated with 6F12 (1:50 dilution) or CR9114 (1:500 dilution) antibody for 1 h at room temperature. After two washes with PBS, secondary antibody goat anti-mouse Alexa Fluor 488 (#A32723) or goat anti-human Alexa Fluor 488 (#A-11013) (1:2000 dilution) was used. After 0.5 h incubation, the samples were washed twice with PBS and then analyzed via FACSCanto II machine. All samples were analyzed with FlowJo software.

### Immunofluorescence assay

MDCK cells or headless HI/19 HA MDCK cells in 24-well plate were fixed with 4% paraformaldehyde for 15 min and then washed with PBS for three times. Next, samples were incubated in 0.1% saponin for 20 min for permeabilization and 5% BSA in 0.1% saponin was added for 3 h blocking. The cells were then stained with primary antibody CR9114 (1:500 dilution) overnight at 4 °C. After three PBS washes, the cells were then incubated with secondary antibody goat anti-human Alexa Fluor 488 (1:2000 dilution) was used for 1 h incubation and then stained with Hoechst 33342 (Invitrogen, H3570) for 5 min. After another three times washes, the samples were imaged via ZOE Fluorescent Cell Imager (Bio-Rad, 1450031).

### ELISA

To compare viral protein levels in normalized headless HI/19 HA virus, 0.1 μg/well BSA, 0.1 μg/well WT HI/19 virus and 0.18 μg/well normalized headless HI/19 HA virus were used to coat ImmunoGrade 96-well plates (BrandTech, #781722) with a carbonate buffer overnight. After three washes with PBS, coated plates were blocked with PBS containing 1% BSA overnight at 4 °C. Then 0.5 μg/ml anti-HA stalk antibody CR9114 (PABL-593), anti-NA antibody (GTX125974) or anti-NP antibody (GTX125989) was gradient diluted and incubated with coated plates overnight. After three washes with PBS, coated plates were incubated with goat anti-rabbit HRP-conjugated antibody (Invitrogen, #31460) or goat anti-human HRP-conjugated antibody (Invitrogen, #31410) for 1 h. After three washes with PBS, coated plates were incubated with 1-Step TMB ELISA Substrate Solutions (Fisher Scientific, PI34028). After 5–10 min incubation, 1M sulfuric acid was added to stop the reaction and plates were read at absorbance 450 nm.

To evaluate antibody response in sera, in-house produced recombinant HI/19 HA head protein and HI/19 HA stalk protein were used. Briefly, HI/19 HA head and HA stalk were fused to a T4 fibrin foldon with His tag to construct expression plasmids. The expression plasmids were transfected into Expi293F cells (Gibco, A14527) and supernatant containing protein was collected after 72 h. Soluble HA head and HA stalk proteins were purified and quantified with BCA assay (Thermo Scientific, A53226). Recombinant HI/19 NA were purchased from Native Antigen (REC31885). WT HI/19 virus or Cal/09 virus was propagated in eggs and concentrated via ultracentrifuge. 0.1 μg/well HI/19 HA head protein, 0.2 μg/well HI/19 HA stalk protein, 0.1 μg/well HI/19 NA protein or 0.1 μg/well whole virus was coated in ImmunoGrade 96-well plates (BrandTech, #781722). After three washes with PBS, coated plates were blocked with PBS containing 1% BSA overnight at 4 °C. Sera samples were serially diluted with PBS containing 1% BSA and incubated in coated plates overnight at 4 °C. In some cases, one or two “blank” uncoated wells per dilution were included in an ELISA plate and plotted to demonstrate the technical background of an assay. For all samples, ELISA signal was developed in the same process as described above.

### ADCC assay

ADCC assays were conducted with Mouse FcγRIV ADCC Bioassay Kit (Promega #M1215). Following the manufacturer’s instruction, MDCK cells plated in a 96-well plate were infected with HI/19 virus (5 MOI). The supernatant was removed 20 hpi and serially diluted sera was added. After 30 min incubation, effector cells were added. After another 6 h incubation, luciferase activity was tested with Bio-Glo Reagent via a plate reader. For HA based ADCC assay, HEK-293T cells were seeded in 96-well plate and the next day cells were transfected with 0.4 μg pLex-HA. After 30 h, the supernatant was removed, and the following steps were the same as described above.

### Microneutralization assay

Sera samples were treated with receptor-destroying enzyme (RDE) (Denka Seiken 370013) for 16 h at 37 °C then and inactivated for 1 h at 56 °C. Serially diluted sera samples were incubated with 1000 PFU HI/19 virus in 96-well plate for 1 hour at 37 °C. 15000 MDCK cells were further seeded into the plate and after 20 h culture the plate was fixed with 4% PFA for 10 min. Next, the cells were stained with CR9114 antibody (0.5 μg/ml) for 4 h and goat anti-human HRP-conjugated antibody for 1 h. Finally, 1-Step TMB ELISA Substrate Solutions was added for 5 min incubation and 1M sulfuric acid was added to stop the reaction. Plates were read at absorbance 450 nm. To calculate 50% inhibitory titer, a four-parameter nonlinear regression was applied using Prism 9 (GraphPad Software) and half-maximal inhibitory concentrations were obtained based on the curve.

### HAI assay

Sera samples were treated with RDE as described above. Serially diluted sera samples were mixed with an equal volume of diluted HI/19 virus (2 HA units) in a v-bottom plate and incubated for 15 min. Chicken blood (1:40 dilution) was further added and after 1 h incubation at 4 °C the results were recorded. HAI titers were calculated based on the highest sera dilution showing HAI.

### NAI assay

Sera samples were treated with RDE as described above. Fetuin (Sigma F3385) was diluted to 25 μg/mL with buffer (KPL coating buffer 50-84-10) and 2.5 μg/well fetuin was coated in ImmunoGrade 96-well plate (BrandTech, #781722). After 24 h, coated plates were washed with PBS-T for three times. Serially diluted sera samples and 0.2 μg recombinant NA (Sino Biological, 40785-V08B-100) were mixed in coated plate. After 18 h incubation at 37 °C, coated plates were washed with PBS-T for three times. Next, 0.1 μg/well peanut agglutinin-HRPO (Sigma A8327) was added. After 2 h incubation, coated plates were washed with PBST for three times. 1-Step TMB ELISA Substrate Solutions (Fisher Scientific, PI34028) was added and after 10 min incubation 1M sulfuric acid was added to stop the reaction. Plates were read at absorbance 450 nm. NAI titers were calculated based on the highest sera dilution showing over 50% inhibition.

### RT-qPCR

Nasal wash samples from infected ferrets were collected. To evaluate viral replication, viral RNA in nasal wash samples was extracted with QIAamp Viral RNA Kits (QIAGEN, 52906). Taqman probe (forward primer: CCTGGAACTGAGAAGCAGATAC, reverse primer: GAATGTAGGCTGCACACTGA, Probe:/56-FAM/AGGACCAGG/ZENAGTGGAGGAAATACCA/3IABkFQ/) targeting viral NP gene were synthesized by IDT. A taqman probe targeting eukaryotic 18S rRNA (Thermo, 4319413E) was the endogenous control. One-step RT-qPCR was conducted with EXPRESS One-Step Superscript qRT-PCR kit (Invitrogen, 11781200) via Applied Biosystems QuantStudio 3 Real-Time PCR System.

### Statistical analysis

All statistical data were shown with Prism 9 (GraphPad Software). Data shown as mean ± SEM. Error bars represent SEM. Statistical analysis was performed using R statistical software (R Foundation for Statistical Computing, Vienna, Austria). For single comparisons, Mann-Whitney U tests were conducted. For experiments with multiple comparisons indicated, wilcoxon rank-sum tests with Bonferroni corrections were conducted and false discovery rate (FDR) adjusted p-values were presented. p < 0.05, *; ns, not significant. Unless otherwise noted, all experiments were performed at least two times and similar results were observed.

## Supporting information

S1 Fig Immunogenicity of a headless HA+WT HI/19 vaccine in mice with pre-existing immunity, related to Fig 4. (A–D) A pre-immune/WT group was immunized separately and the post-vaccination sera were compared to the sera collected from the three vaccine groups described in Fig 4. Post-vaccination sera were used to detect antibody responses against whole HI/19 virus (A), HI/19 HA head (B), HI/19 HA stalk (C) and HI/19 NA (D) via ELISA. N = 5 mice. All experiments were performed at least two times and similar results were observed. For all panels, Mann-Whitney U tests were performed. Data shown as mean ± SEM.
(DOCX)

S1 Table Raw source data associated with the graphs in the manuscript. (XLSX)
