# Supplementary material for: Administration of antigenically distinct influenza viral particle combinations as an influenza vaccine strategy
Source: PLoS Pathog. 2025 Jan 22;21(1):e1012878. doi: 10.1371/journal.ppat.1012878 (PMC11753672; doi:10.1371/journal.ppat.1012878)
Supplement: S1 Fig — (A–D) A pre-immune/WT group was immunized separately and the post-vaccination sera were compared to the sera collected from the three vaccine groups described in Fig 4. Post-vaccination sera were used to detect antibody responses against whole HI/19 virus (A), HI/19 HA head (B), HI/19 HA stalk (C) and HI/19 NA (D) via ELISA. N = 5 mice. All experiments were performed at least two times and similar results were observed. For all panels, Mann-Whitney U tests were performed. Data shown as mean ± SEM. (DOCX) [file ppat.1012878.s001.docx]

**S1 Fig.** **Immunogenicity of headless HA+WT HI/19 vaccine in mice with pre-existing immunity, related to Figure 4. (A-D)** A pre-immune/WT group was immunized separately and compared to the sera collected from the three vaccine groups described in Figure 4. Post-vaccination sera were used to detect antibody responses against whole HI/19 virus **(A)**, HI/19 HA head **(B)**, HI/19 HA stalk **(C)** and HI/19 NA **(D)** via ELISA. N=5 mice. All experiments were performed at least two times and similar results were observed. For all panels, Mann-Whitney U tests were performed. Data shown as mean ± SEM.
